# Preimplantation Genetic Testing of Spinocerebellar Ataxia Type 3/Machado–Joseph Disease—Robust Tools for Direct and Indirect Detection of the *ATXN3* (CAG)_n_ Repeat Expansion

**DOI:** 10.3390/ijms25158073

**Published:** 2024-07-24

**Authors:** Mulias Lian, Vivienne J. Tan, Riho Taguchi, Mingjue Zhao, Gui-Ping Phang, Arnold S. Tan, Shuling Liu, Caroline G. Lee, Samuel S. Chong

**Affiliations:** 1 Preimplantation Genetic Diagnosis Centre, Department of Obstetrics and Gynaecology, National University Hospital, Singapore 119074, Singapore; 2 Department of Paediatrics, Yong Loo Lin School of Medicine, National University of Singapore, Singapore 119228, Singapore; 3 KKIVF Centre, Reproductive Medicine, KK Women’s & Children’s Hospital, Singapore 229899, Singapore; 4 Department of Biochemistry, Yong Loo Lin School of Medicine, National University of Singapore, Singapore 117596, Singapore; 5 Duke-NUS Medical School, Singapore 169857, Singapore; 6 Department of Obstetrics and Gynaecology, Yong Loo Lin School of Medicine, National University of Singapore, Singapore 119228, Singapore; 7 Molecular Diagnosis Centre, Department of Laboratory Medicine, National University Hospital, Singapore 119074, Singapore

**Keywords:** multi-microsatellite haplotyping, preimplantation genetic testing for monogenic disorders, spinocerebellar ataxia type 3/Machado–Joseph disease, triplet-primed PCR

## Abstract

Spinocerebellar ataxia type 3/Machado–Joseph disease (SCA3/MJD) is a neurodegenerative disorder caused by the *ATXN3* CAG repeat expansion. Preimplantation genetic testing for monogenic disorders (PGT-M) of SCA3/MJD should include reliable repeat expansion detection coupled with high-risk allele determination using informative linked markers. One couple underwent SCA3/MJD PGT-M combining *ATXN3* (CAG)_n_ triplet-primed PCR (TP-PCR) with customized linkage-based risk allele genotyping on whole-genome-amplified trophectoderm cells. Microsatellites closely linked to *ATXN3* were identified and 16 markers were genotyped on 187 anonymous DNAs to verify their polymorphic information content. In the SCA3/MJD PGT-M case, the *ATXN3* (CAG)_n_ TP-PCR and linked marker analysis results concurred completely. Among the three unaffected embryos, a single embryo was transferred and successfully resulted in an unaffected live birth. A total of 139 microsatellites within 1 Mb upstream and downstream of the *ATXN3* CAG repeat were identified and 8 polymorphic markers from each side were successfully co-amplified in a single-tube reaction. A PGT-M assay involving *ATXN3* (CAG)_n_ TP-PCR and linkage-based risk allele identification has been developed for SCA3/MJD. A hexadecaplex panel of highly polymorphic microsatellites tightly linked to *ATXN3* has been developed for the rapid identification of informative markers in at-risk couples for use in the PGT-M of SCA3/MJD.

## 1. Introduction

Spinocerebellar ataxia type 3 (SCA3), or Machado–Joseph disease (MJD; OMIM 109150), is a hereditary neurodegenerative disorder that is characterized by progressive cerebellar ataxia, pyramidal and extrapyramidal symptoms, and various neurological manifestations, including neuronal cell death and neurodegeneration [1,2]. It is caused by expansion of a CAG trinucleotide repeat in exon 10 of the *ATXN3* gene on chromosome 14q32.1 [3]. It is the most common autosomal-dominant cerebellar ataxia globally, occurring in 20–50% of families with spinocerebellar ataxia and with varying prevalence rates across different populations [4,5].

Normal *ATXN3* alleles contain 12–44 CAG repeats, while expanded alleles contain ~60–87 repeats [1,6]. Intermediate alleles (45–59 CAG repeats) tend to be unstable and can expand into the pathogenic fully penetrant range in successive generations. Individuals carrying an intermediate allele may exhibit milder forms of ataxia or restless leg syndrome [1,7]. The expanded *ATXN3* CAG repeat exhibits full disease penetrance, although larger expanded alleles are associated with earlier disease onset and faster disease progression [6,7].

Due to the severe and hereditary nature of this disease, at-risk couples with a family history of SCA3/MJD may opt for genetic testing of their preimplantation embryos, which are created through in vitro fertilization (IVF), to select against embryos carrying a repeat expansion. Preimplantation genetic testing for monogenic disorders (PGT-M) allows selection of unaffected embryos for transfer, thus avoiding affected pregnancies and terminations.

PGT-M of SCA3/MJD can be performed by direct interrogation of the CAG repeat locus or by haplotype analysis of linked markers on embryo biopsy samples directly or after whole-genome amplification (WGA). Drüsedau et al. first demonstrated the use of repeat-spanning PCR to determine the inheritance of parental normal *ATXN3* alleles directly on single blastomeres [8]. The embryos were diagnosed as unaffected upon detection of two normal parental *ATXN3* alleles, or as affected otherwise. While successful pregnancy and a live birth were reported, the authors reported a 4.9% allele drop-out (ADO) rate for the repeat-spanning PCR assay during their pre-clinical assay workup and validation studies. Unfortunately, only normal-sized alleles are detected, as expanded *ATXN3* alleles are refractory to standard PCR amplification. Thus, this strategy only works when a couple’s normal alleles are distinguishable. Triplet-primed PCR (TP-PCR) is an alternative method that detects both normal and expanded *ATXN3* alleles, and it can thus be potentially applied to any couple [9,10,11]. Detection of alleles in the expanded range by TP-PCR confirms the diagnosis of SCA3/MJD.

Laitinen-Forsblom et al. recently reported a SCA3/MJD PGT-M case in which multiplex PCR of linked microsatellite markers and genome-wide SNP genotyping were performed on whole-genome-amplified polar body samples [12]. Eleven linked microsatellite markers were identified and the disease-associated haplotype was successfully determined during the clinical cycle despite the high ADO rates of WGA [13,14]. This linkage-based strategy allows exclusion PGT-M to be offered to couples who do not wish to know their own disease status.

While both direct and indirect expanded allele detection strategies have been shown to be effective for the PGT-M of SCA3/MJD independently, a combination of both will increase the diagnostic confidence. Here, we describe a clinical SCA3/MJD PGT-M case involving direct expansion detection via TP-PCR of the *ATXN3* CAG repeat coupled with indirect expansion detection via linked microsatellite marker analysis. Linked microsatellite markers were used to complement the TP-PCR assay due to their compatibility with the multiple displacement amplification method of WGA suitable for downstream TP-PCR, as previously shown for several other repeat expansion disorders [15,16,17]. To simplify the identification of informative markers for linked haplotype analysis in prospective couples who wish to undergo PGT-M for SCA3/MJD, we have also developed a novel single-tube hexadecaplex PCR assay for the simultaneous genotyping of 16 highly polymorphic microsatellite markers flanking the *ATXN3* CAG repeat. 

## 2. Results

### 2.1. Clinical IVF-PGT-M of SCA3/MJD

**Figure 1:**
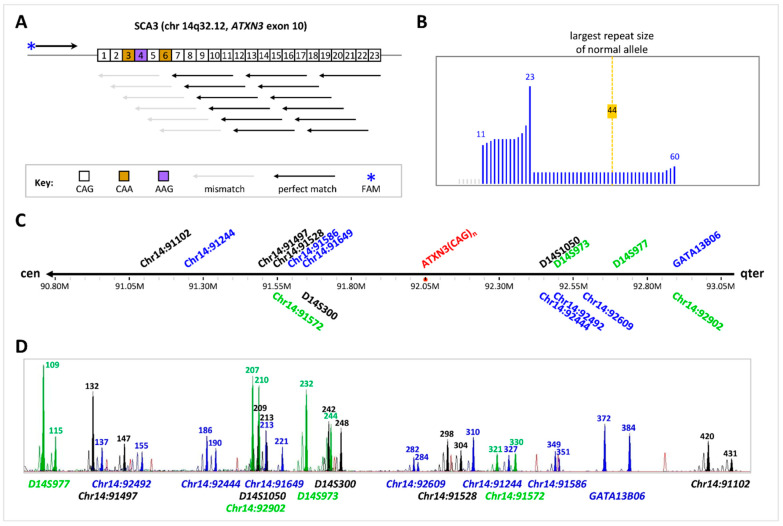
PGT-M of SCA3/MJD by TP-PCR of the *ATXN3* CAG repeat and haplotype analysis of the flanking microsatellite markers. (**A**) *ATXN3* CAG repeat structure and TP-PCR primer annealing positions, (**B**) expected TP-PCR electropherogram pattern of an affected individual, (**C**) relative position of 16 polymorphic microsatellite markers within 1 Mb on either side of the *ATXN3* CAG repeat, and (**D**) representative electropherogram of all 16 markers from 10 ng genomic DNA.

Prior to application to the clinical IVF-PGT-M on the trophectoderm biopsy samples, the SCA3/MJD TP-PCR protocol was developed and optimized on the WGA product of 100 pg of genomic DNA of the affected wife, and it was subsequently validated on the WGA product of single lymphocytes isolated from her peripheral blood. The TP-PCR assay utilizes a gene-specific flanking primer located upstream of the CAG repeat tract, a triplet-primed (TP) primer comprising five CTGs followed by three nucleotides that are complementary to the immediate downstream flanking sequence of the repeat tract and a twenty-nucleotide unique sequence that is identical to the Tail primer (Figure 1A, Table 1).

The 5′ end of every *ATXN3* repeat tract begins with two CAGs, a CAA, an AAG, a CAG, and a CAA, after which the remaining trinucleotides are CAGs. Therefore, the smallest TP-PCR product contains 11 trinucleotides (CAG-CAG-CAA-AAG-CAG-CAA-CAG-CAG-CAG-CAG-CAG), as the TP primer anneals to the first five pure CAGs downstream of the first six trinucleotides. The subsequent TP-PCR products consist of larger fragments successively differing by three base pairs, and they are displayed in a capillary electropherogram as a series of fluorescent peaks of increasing size. The total number of repeats can be determined by counting the number of fluorescent peaks in the electropherogram (Figure 1B). Due to the unique primer design, the last fluorescent peak in the electropherogram is also significantly taller than the few preceding fluorescent peaks to simplify identifying the largest TP-PCR fragment and thus the CAG repeat size of the largest allele in the sample. An SCA3/MJD IVF-PGT-M case was performed using the validated TP-PCR assay for direct *ATXN3* CAG repeat size analysis. A linked marker analysis was performed in parallel to complement the direct repeat expansion analysis and increase the diagnostic confidence.

**Figure 2:**
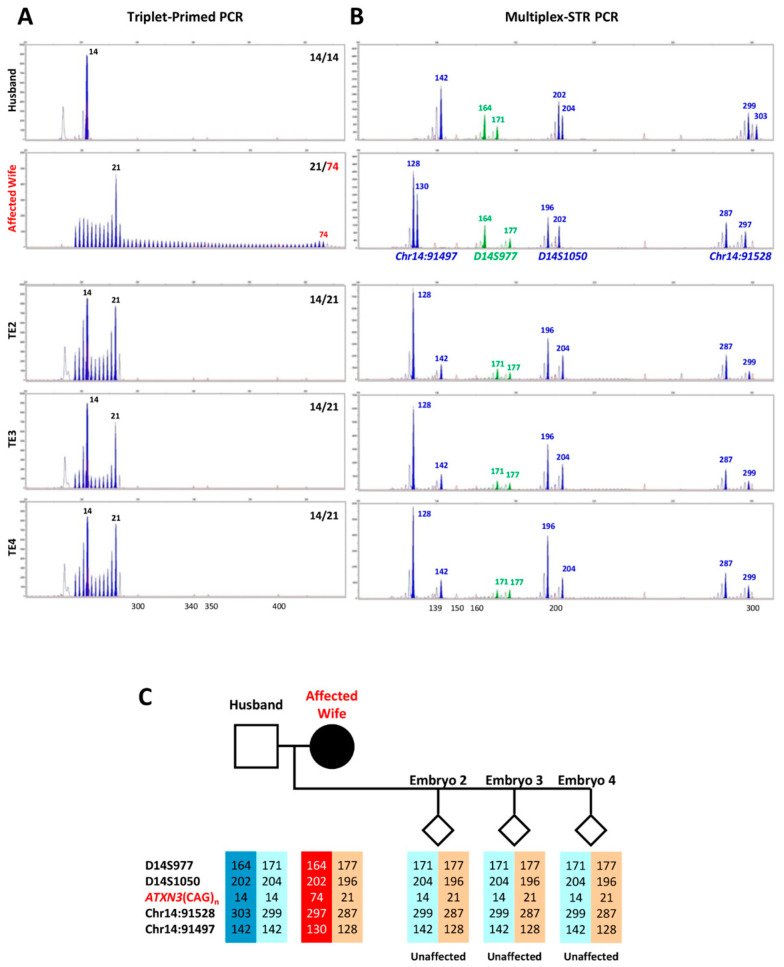
Electropherogram results of the SCA3/MJD clinical IVF-PGT-M case. (**A**) Triplet-primed PCR of the *ATXN3* CAG repeat, and (**B**) multiplex PCR of short tandem repeat markers. The results of the husband and wife were generated from 10 ng genomic DNA, while the embryo results were generated from the whole-genome-amplified product of trophectoderm tissue. (**C**) Inherited paternal and maternal marker haplotypes of embryos. The high-risk maternal haplotype is highlighted in red. The unaffected embryos are indicated by empty diamond symbols.

In the clinical IVF-PGT-M case, the husband was genotyped as being homozygous for 14-repeat normal alleles, while the affected wife had a 21-repeat normal allele and a 74-repeat expanded allele (Figure 2). The TP-PCR results showed that all three embryos inherited the maternal 21-repeat normal allele and the paternal 14-repeat normal allele, indicating that all three embryos were unaffected. The multiplex-marker haplotype analysis showed that all three embryos inherited the maternal low-risk *ATXN3* allele, confirming that all three embryos were unaffected. The frozen–thawed transfer of one of the unaffected blastocyst-stage embryos (#2) successfully resulted in an unaffected live birth.

### 2.2. In Silico Mining of Microsatellite Markers for Hexadecaplex PCR

In order to simplify the process of identifying informative linked markers for SCA3/MJD PGT-M in future prospective couples, we developed a single-tube assay for the simultaneous genotyping of multiple highly polymorphic microsatellites lying within 1 Mb upstream and downstream of the *ATXN3* CAG repeat. Of the 227 markers identified by in silico mining, 139 markers remained after filtering using previously described criteria [18] and manual curation (Supplemental Table S1). Following the preliminary screening of 27 potential markers on 15 to 16 anonymized genomic DNA samples, 11 markers were dropped due to the low PIC values (*Chr14:92416*, *Chr14:92050*, *Chr14:91881*, and *Chr14:91853*), poor amplification results (*Chr14:92691*, *Chr14:91619*, *Chr14:91235*, *Chr14:91121*, and *Chr14:91108*), poor peak patterns and difficulty in allele calling (*Chr14:91142*), or difficulty in amplifying in a highly multiplexed PCR (*Chr14:91068*).

Sixteen potentially highly polymorphic markers that could be multiplexed into a single-tube reaction were selected for inclusion in the single-tube PCR panel. The panel comprises eight upstream (*Chr14:92902*, *GATA13B06*, *D14S977*, *Chr14:92609*, *D14S973*, *Chr14:92492*, *D14S1050*, and *Chr14:92444*) and eight downstream markers (*D14S300*, *Chr14:91649*, *Chr14:91586*, *Chr14:91572*, *Chr14:91528*, *Chr14:91497*, *Chr14:91244*, and *Chr14:91102*). Of these, five are established/published (*GATA13B06*, *D14S977*, *D14S973*, *D14S1050*, and *D14S300*) and eleven are novel (*Chr14:92902*, *Chr14:92609*, *Chr14:92492*, *Chr14:92444*, *Chr14:91649*, *Chr14:91586*, *Chr14:91572*, *Chr14:91528*, *Chr14:91497*, *Chr14:91244*, and *Chr14:91102*) (Figure 1C). The 16 markers provide good redundancy both upstream and downstream of the *ATXN3* CAG repeat, with *Chr14:92444* (~0.4 Mb) and *D14S300* (~0.4 Mb) being the closest upstream and downstream markers, respectively, and *Chr14:92902* (~0.8 Mb) and *Chr14:91102* (~1 Mb) being the farthest upstream and downstream markers, respectively.

A representative capillary electropherogram of the hexadecaplex PCR of GM04866 is shown in Figure 1D. By differentially labelling the primers with different fluorophores tagged to the M13 sequences, each marker can be easily distinguished by a combination of the amplicon peak color and allele size range after capillary electrophoresis. The repeat motifs and primer positions of all 16 markers are listed in Table 2.

### 2.3. Evaluation of Marker Heterozygosity and Polymorphism 

To assess the informativity of the hexadecaplex marker panel, we evaluated the heterozygosity and polymorphism of the 16 markers on a total of 187 DNA samples (from 92 Chinese and 95 Caucasian individuals). The allele frequencies, H_e_, H_o_, and PIC values of each marker were calculated. In total, 176 alleles were observed, where 4 to 17 alleles were observed for each marker and the allele frequencies ranged from 0.005 to 0.51 (Supplemental Figure S1 and Table S2). Population-specific allele sizes were also observed in this study, for example, alleles 285 of *Chr14:92609* and 287 of *Chr14:91528* were only found in the Chinese population, whereas alleles 215 of *D14S1050*, 173 and 189 of *Chr14:92444*, 341 of *Chr14:91586*, 327 of *Chr14:91572*, and 410 of *Chr14:91102* were only found in the Caucasian population.

**Figure 3:**
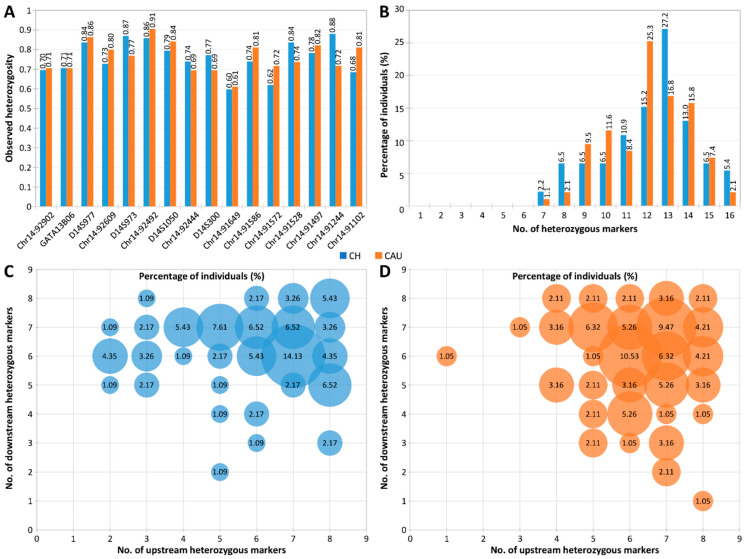
Heterozygosity analysis of 16 microsatellite markers flanking the *ATXN3* CAG repeat in Chinese (*n* = 92) and Caucasian (*n* = 95) population samples. (**A**) Observed heterozygosity of each marker, (**B**) percentage of individuals heterozygous for different numbers of markers, (**C**) percentage of Chinese individuals heterozygous for different numbers of upstream and downstream markers, and (**D**) percentage of Caucasians heterozygous for different numbers of upstream and downstream markers. CH, Chinese; CAU, Caucasian.

The H_e_, H_o_, and PIC value ranges of the 16 markers in the Chinese and Caucasian populations were 0.60–0.85 and 0.65–0.86, 0.60–0.88 and 0.61–0.91, and 0.52–0.83 and 0.60–0.85, respectively (Table 2, Figure 3A). *Chr14:91244* was the most polymorphic in the Chinese population, with an H_o_ of 0.88, whereas *Chr14:92492* was the most polymorphic in the Caucasian population, with an H_o_ of 0.91. Overall, the 16 markers showed remarkable heterozygosity, with 97.8% (90/92) of the Chinese population and 98.9% (94/95) of the Caucasian population heterozygous for at least 8 panel markers and most individuals heterozygous for 12–14 markers (Figure 3B).

More importantly, 100% of Chinese individuals and 97.9% (93/95) of Caucasian individuals were heterozygous for at least two markers on each side of the *ATXN3* CAG repeat (Figure 3C,D). These results demonstrate the high heterozygosity and polymorphism of the hexadecaplex panel in both populations and suggest that sufficient informative markers for linkage-based PGT-M of SCA3/MJD can be identified from genotyping this single marker panel for virtually any prospective couple, without needing to identify additional couple-specific markers.

## 3. Discussion

Like most repeat expansion disorders, the large expanded *ATXN3* alleles are refractory to amplification by standard repeat-flanking PCR. Therefore, SCA3/MJD PGT-M by standard PCR across the *ATXN3* CAG repeat has to rely on detection of the normal non-expanded parental alleles. However, when the affected partner’s normal allele is indistinguishable from the unaffected partner’s normal alleles, this method cannot be used. TP-PCR, on the other hand, is able to detect the presence of the expanded allele directly, overcoming the limitation of standard PCR in being able to identify unaffected embryos only when two normal alleles of different sizes are observed.

Furthermore, due to the unique design of the TP primer, which anneals most strongly when it is in the farthest position from the opposing gene-specific primer, the largest TP-PCR fragments of the two alleles in an embryo allow this method to distinguish between heterozygous and homozygous or compound heterozygous repeat expansion. In an affected embryo with a normal allele and an expanded allele, the largest TP-PCR fragment of the normal allele will appear as a significantly taller fluorescent peak within the normal allele size range, while the largest TP-PCR fragment of the expanded allele will appear as a significantly taller fluorescent peak migrating beyond the normal allele size range. In an affected embryo that is homozygous for two identical-sized expanded alleles, the largest TP-PCR fragments of both expanded alleles should theoretically appear as a significantly taller fluorescent peak beyond the normal allele size range, and at the same time, a significantly taller fluorescent peak will not be observed within the normal allele size range. In an affected embryo that is compound heterozygous for two different-sized expanded alleles, it should be possible to detect two significantly taller fluorescent peaks migrating beyond the normal allele size range, again with no significantly taller fluorescent peak present within the normal allele size range. Thus, unlike standard repeat-flanking PCR, TP-PCR can be offered to most, if not all, couples who seek PGT-M to avoid transmission of the repeat expansion to the next generation, and it has been employed in PGT-M of a number of repeat expansion disorders [15,16,17,19,20,21,22,23,24]. 

The embryo biopsy procedure involves removing a single blastomere from a day-3 cleavage-stage embryo, or a small number of trophectoderm cells from a day-5 blastocyst-stage embryo, for genetic testing. Blastomere biopsy of the cleavage-stage embryos measurably reduces the embryo implantation potential, whereas trophectoderm biopsy of the blastocyst-stage embryos does not similarly reduce the embryo implantation potential [25]. To the best of our knowledge, this is the first reported PGT-M of SCA3/MJD utilizing TP-PCR on whole-genome-amplified embryonic trophectoderm tissue. 

In addition to direct detection of the presence/absence of the expanded allele, we performed multiplex PCR genotyping of four flanking microsatellite markers (two upstream and two downstream of the *ATXN3* CAG repeat) to identify the high- and low-risk maternal haplotypes in the embryos, providing a second separate test to ensure robust PGT-M diagnostic accuracy. Such a genetic testing strategy is highly accurate and reliable because the results from the direct expansion detection (TP-PCR) are corroborated with the indirect linkage analysis (microsatellite marker panel) results. The TP-PCR results of the three embryos analyzed in the clinical IVF-PGT case showed that all three embryos inherited the maternal normal allele, and the multiplex-marker haplotype analysis confirmed that they inherited the maternal low-risk *ATXN3* allele. The clinical IVF-PGT case had a successful embryo transfer and implantation that resulted in a live birth.

We have used the same dual-assay strategy in another clinical SCA3 PGT-M case and successfully detected the repeat expansion in two of the six embryos analyzed by TP-PCR. Both embryos showed a significantly taller fluorescent peak within the normal allele size range and a significantly taller fluorescent peak migrating beyond the normal allele size range, and their affected status was confirmed by linkage analysis.

It is noteworthy that multiple displacement amplification-based WGA has a relatively high ADO rate of ~25% per locus when performed on single blastomeres. ADO could lead to inconclusive results, particularly when it occurs at the CAG repeat locus and when the direct expansion detection is complemented by an insufficient number of closely linked microsatellite markers to identify the high- and low-risk parental haplotypes. When an embryo cannot be unambiguously diagnosed, it may lead to misdiagnosis and loss of perfectly well embryos for transfer. However, the ADO rate can be significantly reduced to 5% per locus with the transitioning of single blastomeres to multi-cell trophectoderm tissue [26]. 

Finally, we developed a multiplex PCR assay for the rapid genotyping of 16 highly polymorphic microsatellite markers located within 1 Mb of the *ATXN3* gene. The hexadecaplex marker panel can be applied to virtually any couple who opts for SCA3/MJD PGT-M and eliminates the need for a customized assay to identify informative markers for each couple. This in turn reduces the pre-clinical assay workup time, which is usually the most time-consuming. For the five previously published/established markers, the observed heterozygosities in our Chinese and Caucasian population samples were comparable to or higher than the heterozygosity values previously observed in the CEPH population [27]. The large number of markers in this panel, with their high observed and expected heterozygosities, provide a very high probability of identifying at least one informative marker on either side of the *ATXN3* CAG repeat that can be used in linkage-based PGT-M for most, if not all, couples at-risk of transmitting SCA3/MJD to their offspring. Coupled with TP-PCR of the *ATXN3* CAG repeat, a combined PGT-M assay of direct and indirect methods for repeat expansion detection will minimize, if not eliminate, most causes of misdiagnosis due to ADO at individual loci or to exogenous DNA contamination. Similar TP-PCR assays and multiplex microsatellite marker panels can be developed for other repeat expansion disorders, including *ATXN1* and *ATXN2* repeat expansions in SCA1 and SCA2, respectively.

We noted that two markers of the same color (*Chr14:91244* and *Chr14:91586*) from the hexadecaplex panel could have overlapping peaks, where the largest 328 bp allele of *Chr14:91244* could overlap with the smallest 329 bp allele of *Chr14:91586*. However, our population screening results indicate that in any single individual, the alleles of one marker do not overlap with the alleles of another marker of the same color. In case of uncertainties in the genotyping results of the two markers, we contend that simplex PCR of the two markers can be performed to verify the genotype in the tested sample. 

Ultimately, this hexadecaplex marker panel can be used for simultaneous genotyping of 16 markers in at-risk couples for the identification of informative markers, thus eliminating the need to customize marker panels for every couple. As all the panel markers are within 1 Mb of the *ATXN3* CAG repeat, the likelihood of recombination between the CAG repeat and any marker is estimated to be <1%. The ESHRE PGT-M Working Group recommended that at least one fully informative microsatellite marker is used at each side of the locus of interest, alongside the detection of the pathogenic variant in few-cell samples after WGA [26]. As the hexadecaplex marker panel has been shown to have at least two heterozygous markers on either side of the *ATXN3* CAG repeat in 100% of the Chinese population and 97.9% of the Caucasian population, the likelihood of needing to customize a linked marker panel PCR after genotyping the hexadecaplex marker panel will be significantly reduced. However, further assessment may be required when using the panel for ethnic groups other than Chinese and Caucasian populations because the heterozygosity values of each marker might differ in other ethnic groups. In the unlikely event that the hexadecaplex marker panel cannot identify sufficient informative markers for a particular at-risk couple, additional markers provided in Supplemental Table S1 can be tested.

In conclusion, robust PGT-M of SCA3/MJD can be achieved by combining direct TP-PCR detection of the expanded *ATXN3* CAG repeat with linkage-based risk allele identification for increased diagnostic confidence. A hexadecaplex panel of highly polymorphic microsatellites tightly linked to *ATXN3* was also developed to simplify the identification of informative markers in at-risk couples for use in PGT-M of SCA3/MJD.

## 4. Materials and Methods

### 4.1. Biological Samples

The genomic DNA of a couple who opted for IVF-PGT-M was extracted and genotyped to confirm the presence of the *ATXN3* CAG repeat expansion in the affected spouse. All the embryos were generated by intracytoplasmic sperm injection of oocytes. Four of eleven recovered oocytes were fertilized and five trophectoderm cells were biopsied from each of the three resultant blastocysts on days 6 and 7 post-fertilization. Each biopsy sample was lysed by adding 1.5 µL of 0.6 mol/L KOH, heated at 65 °C for 10 min, rapidly cooled to 4 °C, and neutralized with 1.5 µL of 0.6 mol/L Tricine. WGA was performed using a GenomiPhi V2 DNA Amplification Kit (Cytiva-Danaher, Buckinghamshire, UK) according to manufacturer’s instructions, except that the incubation time was 4 h. A 2 µL aliquot of WGA product was used as the template in each *ATXN3* TP-PCR and multiplex microsatellite PCR assay. The SCA3/MJD IVF-PGT-M cycle is summarized in Table 3.

The genomic DNA was extracted from up to 16 cell lines purchased from the Coriell Cell Repository (CCR) (Camden, NJ, USA) and used for preliminary screening of potential microsatellite markers and multiplex PCR panel optimization. A total of 92 DNA samples from the unrelated and anonymized cord blood of Chinese babies born at the National University Hospital, Singapore, and 95 Caucasian DNA samples from the Human Variation Panel (HD100CAU, CCR; total 187) were used for determination of the polymorphic information content (PIC) [28], expected heterozygosity (H_e_) [29], and observed heterozygosity (H_o_) [30] of each marker. This study was approved by the Institutional Review Board of the National University of Singapore (07-123E and 13-309E).

### 4.2. SCA3/MJD PGT-M Combining TP-PCR of ATXN3 (CAG)_n_ and Linked Microsatellite Analysis

SCA3/MJD PGT-M was offered to a couple, with the requirement for written informed consent, where the wife had a family history of SCA3 and carried an expanded *ATXN3* allele but was asymptomatic at the time of undergoing this procedure. She was thus at risk of transmitting the expansion to her offspring. The *ATXN3* (CAG)_n_ TP-PCR was initially developed and optimized on the WGA product from 100 pg of genomic DNA from the affected wife, and it was subsequently validated on the WGA product of single lymphocytes isolated from her blood.

The PGT-M TP-PCR assay utilized 0.5 μmol/L *SCA3-F*, 0.05 μmol/L *TP-R*, and 0.5 μmol/L *Tail* primers (Table 1) and was performed in a 50 µL reaction containing 2 U of HotStarTaq DNA polymerase (Qiagen, Hilden, Germany), 1× Q-solution (Qiagen), 1× PCR buffer containing 1.5 mmol/L MgCl_2_ (Qiagen), deoxynucleotide triphosphate (dNTP) mix consisting of 0.2 mmol/L each of dATP, dTTP, dCTP, and dGTP (Roche Applied Science, Penzberg, Germany), and 2 µL of the WGA product of a trophectoderm biopsy sample. The primer *SCA3-F* was fluorescently labelled at the 5′ end with 6-carboxyfluorescein (6-Fam). The thermal cycling involved a 15 min enzyme activation at 95 °C, followed by 35 cycles of denaturation at 98 °C for 45 s, annealing at 60 °C for 1 min, extension at 72 °C for 2 min, and a final extension at 72 °C for 5 min, on the SimpliAmp thermal cycler (Applied Biosystems-Thermo Fisher Scientific, Foster City, CA, USA).

A 2 µL aliquot of the fluorescently labelled PCR product was mixed with 9 μL of Hi-Di™ formamide (Applied Biosystems) and 0.5 μL of GeneScan™ 500 ROX™ dye size standard (Applied Biosystems) before it was denatured at 95 °C for 5 min, rapidly cooled to 4 °C, and resolved across a 36 cm capillary filled with POP7™ polymer in a 3130*xl* Genetic Analyzer (Applied Biosystems). The samples were electrokinetically injected at 1 kV for 15 s and electrophoresed for 40 min at 60 °C. GeneScan analysis was performed using GeneMapper software version 4.0 (Applied Biosystems).

The *ATXN3* TP-PCR analysis was complemented by haplotype analysis of linked microsatellite markers to determine the inheritance of the high- or low-risk maternal allele in each embryo. Markers within 1 Mb upstream and downstream of the *ATXN3* gene were retrieved from the ‘Microsatellite’ and ‘STS Markers’ tracks in the UCSC Genome Browser (https://genome.ucsc.edu/ (accessed on 14 March 2017)). Two upstream (*D14S977* and *D14S1050*) and two downstream (*Chr14:91497* and *Chr14:91528*) markers were informative for the couple and were selected for haplotype analysis. Multiplex PCR of the four microsatellite markers was performed in a 50 µL reaction containing 2.5 U of HotStarTaq DNA polymerase, 1× PCR buffer containing 1.5 mmol/L MgCl_2_, dNTP mix consisting of 0.2 mmol/L each of dATP, dTTP, dCTP, and dGTP, 0.2–0.4 µmol/L of primers (primer sequences and concentrations used are available upon request), and 2 µL of the WGA product of a trophectoderm biopsy sample. One primer from each primer pair was tailed at its 5′ end with either the M13-1 or M13-2 bacteriophage M13 sequence. The thermal cycling involved a 15 min enzyme activation at 95 °C, followed by 30 cycles of denaturation at 98 °C for 45 s, annealing at 60 °C for 1 min, extension at 72 °C for 1 min, and a final extension at 72 °C for 5 min, on the SimpliAmp thermal cycler.

A 2 µL aliquot of each PCR product was subjected to an extension labelling reaction in a 20 µL volume using 0.1 µmol/L each of the 6-Fam-labelled M13-1 primer and Hex-labelled M13-2 primer. The thermal cycling was identical to the multiplex PCR, except that five cycles were performed.

A 1 µL aliquot of the fluorescently labelled PCR product was mixed with 9 μL of Hi-Di™ formamide and 0.3 μL of GeneScan™ 500 ROX™ dye size standard. The samples were prepared for capillary electrophoresis as described above. The samples were electrokinetically injected at 1.2 kV for 23 s and electrophoresed for 20 min at 60 °C. GeneScan analysis was performed using GeneMapper software. Due to the absence of an index case, the maternal high-risk marker haplotype linked to the expanded *ATXN3* allele was established during the actual clinical cycle.

### 4.3. Hexadecaplex Microsatellite Marker Panel Selection

Up to 1 Mb of DNA sequences upstream and downstream of the *ATXN3* gene were downloaded from the National Center for Biotechnology Information (NCBI, http://www.ncbi.nlm.nih.gov/ (accessed on 26 February 2020)) using accession number NC_000014.9 (GRCh38.p13 Primary Assembly). The Tandem Repeat Finder (TRF) DNA analysis program [31] was used to identify microsatellites within the DNA sequences. To determine the preliminary PIC and heterozygosity values of the candidate microsatellites, primer pairs were designed for PCR genotyping, with one primer from each pair tailed at its 5′ end with the M13-1, M13-2, or M13-3 bacteriophage M13 sequence.

The marker genotypes were determined from 15 or 16 cell line DNAs, and those with low PIC and heterozygosity values, poor amplification results, or poor peak pattern were excluded. Sixteen microsatellite markers were eventually selected for the subsequent co-amplification and optimization in a single-tube multiplex reaction (Table 2).

### 4.4. Hexadecaplex Microsatellite PCR and Genotyping

The hexadecaplex microsatellite PCR was performed in a 25 µL reaction containing 2.5 U of HotStarTaq DNA polymerase, 0.75× Q-solution, 1× PCR buffer containing 1.5 mmol/L MgCl_2_, dNTP mix consisting of 0.2 mmol/L each of dATP, dTTP, dCTP, and dGTP, 0.2–0.5 µmol/L of primers (Table 2), and 10 ng of genomic DNA. The thermal cycling was identical to the PGT-M microsatellite multiplex PCR, except that the extension was performed at 72 °C for 1 min with an increment of 6 s after every cycle.

A 2 µL aliquot of the PCR product was subjected to extension labelling in a 20 µL reaction using 0.2 µmol/L each of the 6-Fam-labelled M13-1 primer, Hex-labelled M13-2 primer, and Ned-labelled M13-3 primer. The thermal cycling was identical to the hexadecaplex PCR, except that 10 cycles were performed and the extension was held at 72 °C for 1 min at each cycle.

The capillary electrophoresis conditions were identical to the multiplex microsatellite PCR of the IVF-PGT-M case. The allele frequency, PIC, H_e_, and H_o_ values of each marker were calculated using Microsoft Excel.

## Figures and Tables

**Table 1:** *ATXN3* (CAG)_n_ TP-PCR primers and expected product sizes.

Primer	Primer Sequence (5′-3′)	GenBank ID: Nucleotides	Concentration (µmol/L)	Expected TP-PCR Product Size
*SCA3-F*	6-Fam-ATGTCTAGATTTCCTAAGATCAGCACTTCC	NG_008198.2: 40371–40400	0.50	240 bp + (CAG)_n_
*TP-R*	GTTTCGGCGTTACGAGTGGA**CCC**CTGCTGCTGCTGCTG	-	0.05	
*Tail*	GTTTCGGCGTTACGAGTGGA	-	0.50	

Bolded and underlined nucleotides are complementary to the immediate downstream flanking sequence of the repeat locus.

**Table 2:** Hexadecaplex microsatellite marker PCR details.

Microsatellite Marker	Repeat Motif	Primer Sequence (5′-3′) ^a^		Concentration (µmol/L)	Amplicon Size Range (bp) ^f^	H_e_^f^		H_o_^f^		PIC ^f^	
						CH	CAU	CH	CAU	CH	CAU
***Chr14:92902***	(GAG)_n_	F	^M13−2^GCTAAGACCAAAATGTGAGCCAG	0.3	190–213	0.64	0.69	0.70	0.71	0.57	0.64
		R	^b^CCCATGAATTTCGAGTGTGTAAGG	0.3							
*GATA13B06*	(CTAT)_n_	F	^M13−1^CAGACAGCCATGGTGGTTCTTG	0.3	368–402	0.75	0.72	0.71	0.71	0.71	0.67
		R	^c^TGTAAGGGTCTGGATGATTCTGG	0.3							
*D14S977*	(CA)_n_	F	^M13−2^CTTATAGGATCTGGGGTGTACGC	0.2	102–128	0.84	0.85	0.84	0.86	0.82	0.84
		R	^d^TTTCTCTCCATGCACTCCCTG	0.2							
***Chr14:92609***	(AC)_n_	F	^M13−1^CCAGCGCCTTGATCTTGGAAG	0.3	263–287	0.78	0.78	0.73	0.80	0.75	0.75
		R	^b^ACTGTCTCCGCTTACAGAAGTTG	0.3							
*D14S973*	(AC)_n_	F	^M13−2^CCAGCAAAGGACAGTTCTGC	0.3	218–254	0.83	0.81	0.87	0.77	0.81	0.78
		R	^b^AGCCGGAAGAATGGAGAATAGC	0.3							
***Chr14:92492***	(TG)_n_	F	^M13−1^TTTCCAAGGCTCCTATGGACCC	0.2	126–158	0.85	0.86	0.86	0.91	0.83	0.85
		R	^b^CCGTGCTTTAACCCTCTACCC	0.2							
*D14S1050*	(GT)_n_	F	^M13−3^TCTCTTAGGGCACCTGTGG	0.3	193–215	0.77	0.82	0.79	0.84	0.73	0.80
		R	^b^CATTGCTGGGGCAAGGTAAGG	0.3							
***Chr14:92444***	(GT)_n_	F	^M13−1^GCCTCCCCTTATTCATGGAGTAG	0.2	169–191	0.70	0.72	0.74	0.69	0.64	0.69
		R	^b^CCTCCAACAACACTTGCACACC	0.2							
*D14S300*	(GT)_n_	F	^M13−3^TACTCTGCCACAGACACCTTC	0.3	238–252	0.72	0.74	0.77	0.69	0.67	0.69
		R	^b^ACCTTACTAAAGGGCTGCCATG	0.3							
***Chr14:91649***	(AC)_n_	F	^M13−1^CAGCCGGGGCAACAATCTC	0.2	213–225	0.60	0.65	0.60	0.61	0.52	0.60
		R	^b^GGTGATAACTGTAGACCAGCTC	0.2							
***Chr14:91586***	(TC)_n_	F	^M13−1^CCTCCTTTTGGCTGTTTGAACTG	0.2	329–361	0.75	0.83	0.74	0.81	0.71	0.81
		R	^e^TTATTGGAGCTGGCCTTAGAGC	0.2							
***Chr14:91572***	(GT)_n_	F	^M13−2^GAACTCAAAATAAGAGGCTTTGGGG	0.5	317–331	0.68	0.73	0.62	0.72	0.63	0.70
		R	^b^GGCAGCAAGCTCTTCATGTTAC	0.5							
***Chr14:91528***	(AC)_n_	F	^M13−3^GGGTCTGTTAGGAAGTGAGATAAGG	0.3	285–317	0.76	0.78	0.84	0.74	0.73	0.75
		R	^b^GCTAAAGTGACCCTCTTGCCTC	0.3							
***Chr14:91497***	(GT)_n_	F	^b^CAATGTCTTTCTCAGATGTGTGGG	0.2	122–154	0.71	0.75	0.78	0.82	0.68	0.72
		R	^M13−3^CTCTCCAATGAAACAGATGCCC	0.2							
***Chr14:91244***	(TG)_n_	F	^M13−1^CAACTTCATAGCTCATGACCTGC	0.2	310–328	0.79	0.71	0.88	0.72	0.76	0.68
		R	^b^AGGCTCAAACTCACACAGTCAG	0.2							
***Chr14:91102***	(AC)_n_	F	^d^TTTCAAGATCAAGAACGGAGGGG	0.4	410–446	0.72	0.86	0.68	0.81	0.67	0.85
		R	^M13−3^CATCTGCTTCTGGGGATTGGAG	0.4							

Markers highlighted in bold are novel. ^a^ Primer sequences were based on genome assembly build GRCh38/hg38. ^b^ 5′ GTTT tailed; ^c^ 5′ GTT tailed; ^d^ 5′ G tailed; ^e^ 5′ GT tailed. ^M13−1^, 5′ GGTTTTCCCAGTCACGAC tailed; ^M13−2^, 5′ GTAAAACGACGGCCAGTG tailed; ^M13−3^, 5′ CATGGTCATAGCTGTTTCCTG tailed. ^f^ Amplicon size, H_e_ (expected heterozygosity), H_o_ (observed heterozygosity), and PIC (polymorphism information content) were determined from 92 CH (Chinese) and 95 CAU (Caucasian) DNA samples.

**Table 3:** Outcome of IVF-PGT-M for SCA3/MJD.

Oocytes recovered	11
Oocytes fertilized with two pronuclei	4
Embryos biopsied	3
Unaffected embryos	3
Embryos transferred during the same cycle	0
Embryos frozen	3
Frozen thawed embryos transferred at a subsequent cycle	1
Positive hCG	1
Pregnancy with fetal heartbeat	1
Live birth	1
Affected embryos	0
Embryos with no diagnosis	0

PGT-M, preimplantation genetic testing for monogenic disorder; SCA3/MJD, spinocerebellar ataxia type 3/Machado–Joseph disease; hCG, human chorionic gonadotropin.

## Data Availability

The original contributions presented in this study are included in the article/Supplementary Materials; further inquiries can be directed to the corresponding author.

## Supplementary Materials

The following supporting information can be downloaded at: https://www.mdpi.com/article/10.3390/ijms25158073/s1.
