# Supplementary material for: Preimplantation Genetic Testing of Spinocerebellar Ataxia Type 3/Machado–Joseph Disease—Robust Tools for Direct and Indirect Detection of the ATXN3 (CAG)n Repeat Expansion
Source: Int J Mol Sci. 2024 Jul 24;25(15):8073. doi: 10.3390/ijms25158073 (PMC11311680; doi:10.3390/ijms25158073)
Supplement: Supplementary file 1 [file ijms-25-08073-s001.zip › SCA3 TPnSTRPGD TableS1 7Jun24_IJMS.pdf]

Supplemental Table S1. *In silico* mining of microsatellite markers located approximately 1 Mb upstream and 1 Mb downstream of the *ATXN3* CAG on chromosome 14q32.12, identified using Tandem Repeat Finder (TRF)

Microsatellite marker[a]	Marker location[b]	Repeat motif	No. of repeats	Percentage of matches[c]	Score[d]
Chr14:93100	93100573-93100527	TC	24	86	69
Chr14:93066	93066341-93066307	AC	17.5	93	61
Chr14:93030	93030469-93030432	AATG	9.5	88	58
Chr14:93001	93001327-93001297	GTTT	8	92	55
Chr14:92931	92931603-92931579	CCT	8.3	100	50
Chr14:92913	92913525-92913479	AAT	15.7	100	94
Chr14:92910	92910734-92910697	AATA	9.8	82	51
**Chr14:92902**	**92902409-92902358**	**GAG**	**17.7**	**92**	**88**
**GATA13B06 (CHLC.GATA142D12/D14S559)**	**92896240-92896165**	**CTAT**	**19**	**91**	**127**
Chr14:92887	92887465-92887418	TCCA	12	81	51
Chr14:92816	92816580-92816523	AGAAG	11	88	53
Chr14:92808	92808274-92808245	TTG	10	92	51
Chr14:92765	92765855-92765807	AC	24.5	100	98
Chr14:92756	92756502-92756462	GTTT	11	85	61
**D14S977 (AFMA116ZF5)**	**92703100-92703057**	**CA**	**22**	**100**	**88**
Chr14:92696	92696788-92696755	CCTT	8.5	86	50
Chr14:92695	92695975-92695932	GAGG	10.8	80	52
	92695927-92695897	GAAG	7.8	92	53
Chr14:92691	92691978-92691944	GT	17.5	100	70
Chr14:92678	92678488-92678451	GTGG	9.5	88	58
Chr14:92672	92672689-92672651	AC	19.5	89	60
Chr14:92661	92661759-92661724	TG	18	100	72
Chr14:92633	92633895-92633857	CA	19.5	89	60
Chr14:92629	92629154-92629118	TC	18.5	100	74
**Chr14:92609**	**92609898-92609844**	**AC**	**27.5**	**92**	**92**
Chr14:92592	92592691-92592643	AAT	16.3	100	98
Chr14:92570	92570355-92570322	GT	17	93	59
Chr14:92561	92561173-92561105	TA	34.5	82	75
**D14S973 (AFMA112XC1)**	**92518826-92518790**	**AC**	**18.5**	**100**	**74**
**Chr14:92492**	**92493018-92492960**	**TG**	**29.5**	**100**	**118**
Chr14:92488	92488066-92487966	AGG	32.3	82	98
Chr14:92486	92486537-92486509	AAAAC	6	92	51
Chr14:92474	92474864-92474794	AT	35.5	88	52
Chr14:92468	92468935-92468893	AC	21.5	100	86
**D14S1050 (AFM343VF1)**	**92449323-92449282**	**GT**	**21**	**100**	**84**
AFMB308ZE9	92448380-92448346	TG	17.5	100	70
**Chr14:92444**	**92444972-92444904**	**GT**	**34**	**88**	**93**
Chr14:92440	92440882-92440854	CCCTT	5.8	100	58
Chr14:92426	92426017-92425981	ATTG	9.3	93	65
Chr14:92416	92416152-92416102	AC	26	90	77
Chr14:92408	92408202-92408167	AC	18	100	72

Chr14:92407	92407900-92407858	AC	21.5	100	86
Chr14:92395	92395466-92395433	GT	17	100	68
Chr14:92373	92373212-92373172	TG	20.5	94	73
Chr14:92372	92372998-92372940	AG	29.5	85	64
Chr14:92372	92372919-92372868	AAGG	13	100	104
Chr14:92351	92351951-92351877	TTCCT	16.4	86	92
Chr14:92351	92351258-92351219	TG	20	89	62
Chr14:92343	92343716-92343581	AAAG	33.8	93	146
Chr14:92342	92342399-92342364	AAAT	9	100	72
Chr14:92334	92334930-92334867	GAT	21.3	90	101
Chr14:92316	92316225-92316166	TTTTG	11.6	82	75
Chr14:92294	92294408-92294366	TAT	14.3	100	86
Chr14:92291	92291142-92291109	CCAT	8.5	93	59
Chr14:92273	92273231-92273193	TTTG	10	86	53
AFM214YE5	92269884-92269835	GT	25	100	100
CHLC.ATA29D09	92265595-92265561	ATA	11.7	100	70
Chr14:92262	92262035-92261997	TG	19.5	100	78
Chr14:92252	92252949-92252879	CT	35.5	82	88
Chr14:92252	92252799-92252744	CA	28	92	94
Chr14:92251	92251088-92250890	TTCT	50	88	199
Chr14:92173	92173446-92173303	AT	72	83	162
Chr14:92102	92102275-92102197	CTTT	19	84	77
Chr14:92099	92099980-92099862	TTTC	29.5	82	118
Chr14:92078	92078379-92078348	AAAT	8.5	86	50
ATXN3 (CAG)n	92071052-92071011	CAG	14	84	57
Chr14:92060	92060271-92060222	AT	25	91	64
Chr14:92050	92050194-92050144	AC	26	84	68
Chr14:92042	92042977-92042947	TA	15.5	100	62
Chr14:92031	92031143-92031094	AATA	12.5	91	55
Chr14:91981	91981013-91980980	AT	17.5	87	52
Chr14:91948	91948322-91948294	AAAC	7.3	100	58
Chr14:91929	91929128-91929081	TG	24.5	93	80
Chr14:91922	91922358-91922308	TTTA	12.5	95	93
Chr14:91919	91919435-91919391	TCCT	11.3	100	90
	91919392-91919331	TTTCT	13.6	90	82
Chr14:91914	91914849-91914817	AAAAC	6.8	96	59
Chr14:91885	91885627-91885598	CAAC	7.5	92	51
Chr14:91881	91881154-91881121	GT	17	100	68
Chr14:91880	91880562-91880517	AC	23	90	74
Chr14:91853	91853690-91853650	TG	20.5	100	82
Chr14:91845	91845273-91845233	TATT	10.3	100	82
Chr14:91803	91803429-91803381	TG	24.5	82	62
Chr14:91791	91791237-91791141	CCCTT	19.6	84	81
Chr14:91746	91746611-91746532	AT	40	87	61
GGAA21G11	91736529-91736502	CCTT	7	100	56
AFM304YA5 (SHGC-1415)	91716766-91716713	GT	27	92	90
Chr14:91702	91702706-91702676	TG	15.5	100	62
Chr14:91672	91672300-91672262	AAAC	9.8	88	51

Chr14:91672	91672054-91672023	TTG	10.7	100	64
**D14S300**	**91660145-91660117**	**GT**	**14.5**	**100**	**58**
**Chr14:91649**	**91649362-91649332**	**AC**	**15.5**	**100**	**62**
Chr14:91619	91619912-91619862	AC	25.5	100	102
Chr14:91612	91612071-91612013	AAAG	14.8	96	109
Chr14:91595	91595332-91595291	AAAT	10.8	89	68
**Chr14:91586**	**91586594-91586547**	**TC**	**24**	**95**	**87**
**Chr14:91572**	**91572972-91572941**	**GT**	**16**	**100**	**64**
Chr14:91569	91569440-91569408	AGGA	8.3	93	57
Chr14:91545	91545543-91545466	AAGG	19.5	80	70
Chr14:91544	91544579-91544548	TTCT	8.5	93	50
Chr14:91543	91543890-91543864	ATATT	5.4	100	54
AFM030XH4	91537388-91537360	GT	14.5	100	58
	91537360-91537326	GA	17.5	87	52
**Chr14:91528**	**91528421-91528379**	**AC**	**21.5**	**100**	**86**
Chr14:91517	91517488-91517448	AT	20.5	94	73
Chr14:91504	91504361-91504326	TTTTC	7.8	96	51
**Chr14:91497**	**91497379-91497342**	**GT**	**19**	**100**	**76**
Chr14:91479	91479329-91479294	AC	18	100	72
Chr14:91472	91472851-91472825	TTGG	6.8	100	54
Chr14:91421	91421976-91421941	AAAT	8.8	93	63
Chr14:91366	91366202-91366154	TG	24.5	100	98
Chr14:91360	91360291-91360253	GT	19.5	100	78
Chr14:91345	91345191-91345167	AT	12.5	100	50
Chr14:91290	91290298-91290271	TTGTT	5.6	100	56
Chr14:91259	91259638-91259600	TGTTT	7.2	88	51
**Chr14:91244**	**91244257-91244213**	**TG**	**22.5**	**95**	**81**
Chr14:91242	91242541-91242516	CCAC	6.5	100	52
Chr14:91235	91235484-91235437	TTAT	12	100	96
Chr14:91213	91213720-91213692	AACA	7.3	100	58
Chr14:91205	91205796-91205755	AGA	14	100	84
D14S593	91202085-91202059	ATT	9	100	54
Chr14:91184	91184687-91184615	TTCC	18	91	56
	91184575-91184551	CTTT	6.3	100	50
Chr14:91183	91183007-91182899	AAAAT	21.4	83	125
Chr14:91181	91181996-91181966	AAAT	7.8	100	62
Chr14:91181	91181675-91181624	AAAG	13.8	80	58
Chr14:91177	91177019-91176947	GAAA	18.8	82	55
Chr14:91157	91157332-91157011	ATCC	80.5	84	367
Chr14:91142	91142882-91142804	GT	39	85	99
Chr14:91121	91121654-91121610	ATT	15	100	90
Chr14:91117	91117686-91117642	GT	22.5	90	72
AFMA238WA9	91111291-91111249	ATAT	10.8	84	50
	91111249-91111203	AC	23.5	100	94
Chr14:91110	91110433-91110407	AAAG	6.8	100	54
Chr14:91108	91108565-91108527	CT	19.5	94	69
	91108527-91108488	CA	20	100	80
**Chr14:91102**	**91102786-91102737**	**AC**	**25**	**95**	**91**

Chr14:91093	91093820-91093796	TGTT	6.3	100	50
Chr14:91091	91091187-91091145	TGTAT	8.2	80	50
Chr14:91078	91078919-91078860	AAAT	15.8	86	90
Chr14:91077	91077442-91077354	CTTC	22	85	72
Chr14:91077	91077284-91077255	TC	15	92	51
Chr14:91074	91074161-91074119	TG	21.5	100	86
Chr14:91068	91068116-91068060	GT	28.5	92	87
Chr14:91062	91062611-91062559	TA	26.5	100	106

[a]Established markers are named as published. Novel markers are named with Chr14 prefix followed by their distance (in 10^3 base pairs) from chromosome 14pter, based on the UCSC genome browser reference sequence (GRCh38/hg38).

[b]Base pairs from chromosome 14pter, based on GRCh38/hg38.

[c]Overall percentage of matches between adjacent copies as calculated by TRF (http://tandem.bu.edu/trf/trf.html).

[d]Alignment score as calculated by TRF with settings as +2 for a base pair match and -7 for a mismatch or an indel in the repeat stretch. Alignment score is the weight for match, mismatch and indels.

Black-shaded marker is the ATXN3 CAG repeat; bolded markers were included in the final hexadecaplex panel; gray-shaded markers were excluded after preliminary screening due to low PIC values, poor amplification results, or poor peak pattern and difficulty in allele calling.
