# Supplementary material for: Preimplantation Genetic Testing of Spinocerebellar Ataxia Type 3/Machado–Joseph Disease—Robust Tools for Direct and Indirect Detection of the ATXN3 (CAG)n Repeat Expansion
Source: Int J Mol Sci. 2024 Jul 24;25(15):8073. doi: 10.3390/ijms25158073 (PMC11311680; doi:10.3390/ijms25158073)
Supplement: Supplementary file 1 [file ijms-25-08073-s001.zip › SCA3 TPnSTRPGD TableS2 7Jun24_IJMS.pdf]

Supplemental Table S2. Allele frequency distribution of the 16 microsatellite markers flanking *ATXN3* $(CAG)_n$

Allele No.	Chr14:92902			GATA13B06			D14S977			Chr14:92609		
	Allele	Frequency		Allele	Frequency		Allele	Frequency		Allele	Frequency	
		CH	CAU		CH	CAU		CH	CAU		CH	CAU
1	190	0.47	0.44	368		0.01	102		0.01	263		0.01
2	207	0.35	0.17	372	0.04	0.03	104	0.10	0.06	265	0.36	0.35
3	210	0.05	0.25	376	0.38	0.39	106	0.01	0.08	267	0.01	0.02
4	213	0.13	0.14	380	0.28	0.32	108	0.08	0.30	269	0.01	0.19
5				384	0.14	0.15	110	0.10	0.07	271	0.00	0.01
6				388	0.01	0.02	112	0.01	0.02	273	0.01	0.00
7				390	0.01	0.00	114	0.09	0.07	275	0.03	0.03
8				394	0.10	0.06	116	0.05	0.05	277	0.16	0.02
9				398	0.04	0.02	118	0.04	0.07	279	0.13	0.18
10				402	0.01		120	0.33	0.14	281	0.03	0.07
11							122	0.08	0.09	283	0.20	0.12
12							124	0.09	0.04	285	0.08	0.00
13							126	0.01		287		0.01
14							128	0.01				

Allele No.	D14S973			Chr14:92492			D14S1050			Chr14:92444		
	Allele	Frequency		Allele	Frequency		Allele	Frequency		Allele	Frequency	
		CH	CAU		CH	CAU		CH	CAU		CH	CAU
1	218		0.01	126	0.01	0.00	193	0.01		169	0.01	0.01
2	230	0.01	0.02	128	0.01	0.01	203	0.24	0.27	173	0.00	0.08
3	232	0.26	0.26	130	0.01	0.00	205	0.03	0.05	177	0.00	0.01
4	234	0.01	0.02	132	0.09	0.27	207	0.13	0.11	179	0.04	0.03
5	236	0.03	0.01	134	0.14	0.06	209	0.34	0.23	181	0.37	0.47
6	238	0.01	0.01	136	0.11	0.05	211	0.20	0.12	183	0.26	0.18
7	240	0.09	0.04	138	0.27	0.14	213	0.05	0.14	185	0.32	0.06
8	242	0.11	0.25	140	0.19	0.08	215		0.08	187	0.02	0.09
9	244	0.23	0.22	142	0.06	0.05				189		0.07
10	246	0.15	0.12	144	0.02	0.11				191		0.01
11	248	0.06	0.03	146	0.01	0.06						
12	250	0.02	0.02	148	0.03	0.12						
13	252	0.02	0.00	150	0.01	0.05						
14	254		0.01	152	0.02	0.01						
15				154	0.01	0.00						
16				156	0.02	0.00						
17				158	0.01	0.00						

Allele No.	D14S300			Chr14:91649			Chr14:91586			Chr14:91572		
	Allele	Frequency		Allele	Frequency		Allele	Frequency		Allele	Frequency	
		CH	CAU		CH	CAU		CH	CAU		CH	CAU
1	238	0.01		213	0.51	0.51	329		0.02	317	0.02	0.03
2	240	0.01		215	0.00	0.02	341		0.18	319	0.04	0.09

3	242	0.29	0.32	219	0.36	0.28	345	0.04	0.12	321	0.35	0.05
4	244	0.00	0.02	221	0.09	0.08	347	0.02	0.18	323	0.08	0.03
5	246	0.37	0.28	223	0.03	0.09	349	0.03	0.09	325	0.01	0.16
6	248	0.13	0.27	225	0.01	0.02	351	0.33	0.04	327	0.00	0.17
7	250	0.20	0.11				353	0.14	0.02	329	0.43	0.45
8	252		0.01				355	0.34	0.27	331	0.08	0.02
9							357	0.03	0.02			
10							359	0.07	0.06			
11							361	0.01				

**Allele No.**	**Chr14:91528**			**Chr14:91497**			**Chr14:91244**			**Chr14:91102**		
	**Allele**	**Frequency**		**Allele**	**Frequency**		**Allele**	**Frequency**		**Allele**	**Frequency**	
		**CH**	**CAU**		**CH**	**CAU**		**CH**	**CAU**		**CH**	**CAU**
1	285	0.01	0.01	122		0.01	310	0.18	0.16	410		0.07
2	287	0.04	0.00	126		0.01	312	0.05	0.07	412	0.01	0.01
3	291	0.01	0.04	128	0.02	0.00	314	0.05	0.16	416	0.02	0.01
4	293	0.02	0.01	132	0.49	0.41	316	0.27	0.48	418	0.02	0.11
5	295	0.39	0.36	134	0.03	0.24	318	0.30	0.07	420	0.02	0.07
6	297	0.03	0.06	136	0.00	0.04	320	0.06	0.02	422	0.35	0.11
7	299	0.02	0.19	138	0.01	0.09	322	0.03	0.01	424	0.36	0.26
8	301	0.03	0.08	140	0.05	0.02	324	0.01	0.02	426	0.16	0.15
9	303	0.06	0.19	142	0.06	0.12	326	0.02	0.01	428	0.04	0.05
10	305	0.27	0.02	144	0.11	0.03	328	0.03	0.01	430	0.01	0.11
11	307	0.08	0.02	146	0.17	0.04				432	0.01	0.05
12	309	0.03	0.01	148	0.02					434	0.01	
13	311	0.01	0.01	150	0.02					446	0.01	
14	313	0.01		152	0.01							
15	317	0.01		154	0.01							
