# Supplementary figures and images for: Preimplantation Genetic Testing of Spinocerebellar Ataxia Type 3/Machado–Joseph Disease—Robust Tools for Direct and Indirect Detection of the ATXN3 (CAG)n Repeat Expansion
Source: Int J Mol Sci. 2024 Jul 24;25(15):8073. doi: 10.3390/ijms25158073 (PMC11311680; doi:10.3390/ijms25158073)

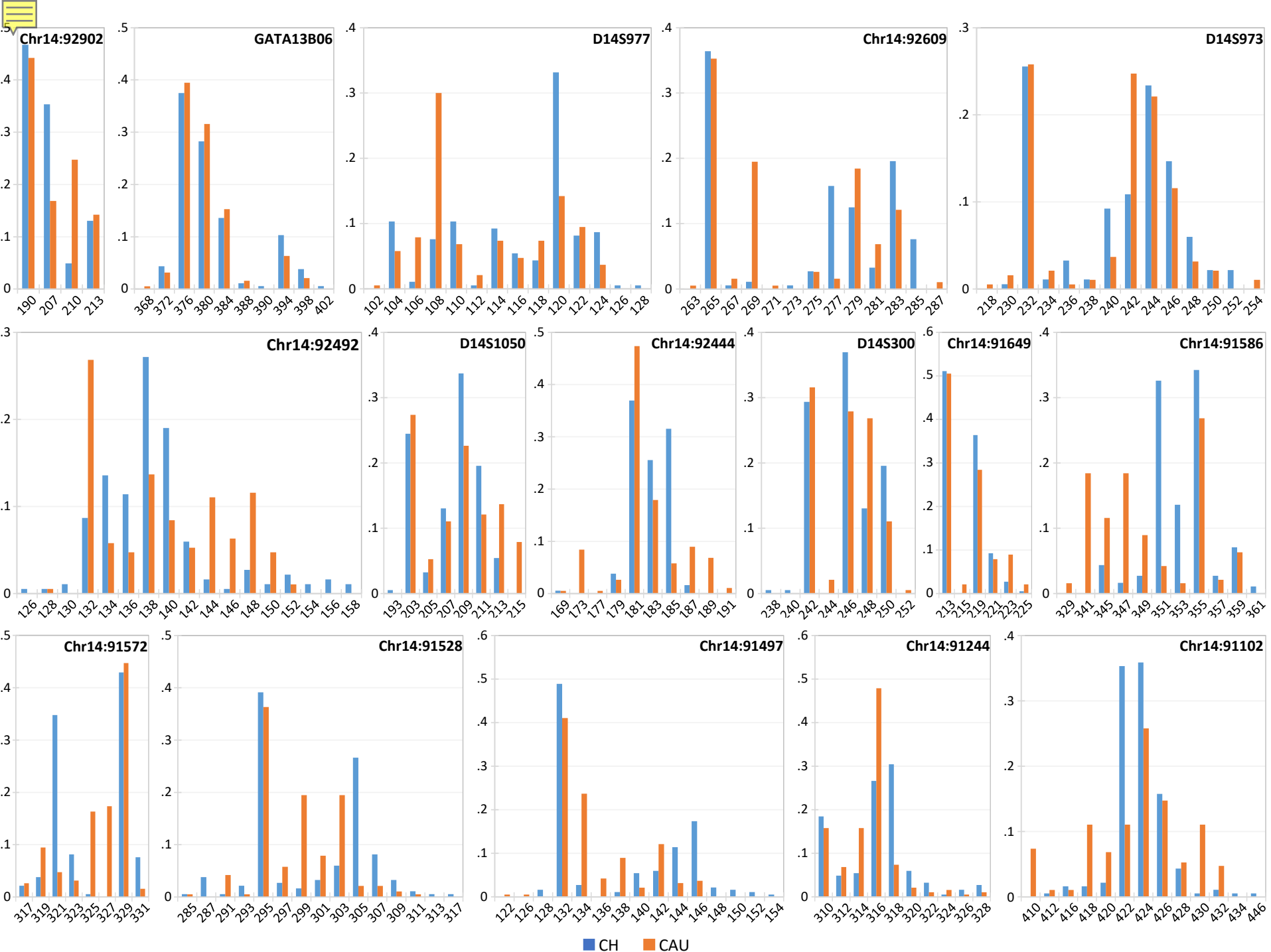

Supplement: Supplementary file 1 [file ijms-25-08073-s001.zip › SCA3 TPnSTRPGD FigS1 28Apr24_TM.pdf]
